# Affinity of Alkylphosphocholines to Biological Membrane of Prostate Cancer: Studies in Natural and Model Systems

**DOI:** 10.1007/s00232-014-9674-8

**Published:** 2014-05-22

**Authors:** Anita Wnętrzak, Ewelina Lipiec, Kazimierz Łątka, Wojciech Kwiatek, Patrycja Dynarowicz-Łątka

**Affiliations:** 1 M. Smoluchowski Institute of Physics, Jagiellonian University, Reymonta 4, 30-059 Kraków, Poland; 2 The Henryk Niewodniczański Institute of Nuclear Physics (PAN), 31-342 Kraków, Poland; 3 Faculty of Chemistry, Jagiellonian University, Ingardena 3, 30-060 Kraków, Poland

**Keywords:** Alkylphosphocholines, Model membrane, Langmuir monolayers, Prostate cancer

## Abstract

The effectiveness of two alkylphosphocholines (APCs), hexadecylphosphocholine (miltefosine) and erucylphosphocholine to combat prostate cancer has been studied in vitro with artificial cancerous membrane, modelled with the Langmuir monolayer technique, and on cell line (Du-145). Studies performed with the Langmuir method indicate that both the investigated drugs have the affinity to the monolayer mimicking prostate cancer membrane (composed of cholesterol:POPC = 0.428) and the drug-membrane interactions are stronger for erucylphosphocholine as compared to hexadecylphosphocholine. Moreover, both studied drugs were found to fluidize the model membrane, which may lead to apoptosis. Indeed, biological studies confirmed that in Du-145 cell line both investigated alkylphosphocholines cause cell death primarily by apoptosis while necrotic cells constitute only a small percentage of APC-treated cells.

## Introduction

There are plethora of studies confirming that many chemicals, including drugs, dietary supplements or environmental pollutants affect cellular membrane properties, which can further influence on cell functioning. Most frequently observed effects include changes in membrane fluidity, permittivity or membrane lipids organisation/composition as recently reviewed by Tekpli et al. (2013). In consequence, membrane function can be altered, resulting either in improved cells functioning or their death.

Changes in membrane properties observed in vivo can be verified in model systems, which are advantageous—as compared to natural ones—of being simple and well-defined, contrary to highly variable and complex living systems. Moreover, models enable a systematic analysis of a selected phenomenon of interest, while living systems provide only a global view on a particular problem. Among various membrane models applied (reviewed in Hąc-Wydro and Dynarowicz-Łątka 2008; Chan and Boxer 1999), most popular are Langmuir monolayers (Hąc-Wydro and Dynarowicz-Łątka 2008; Maget-Dana 1999) and liposomes/vesicles (Kell 1981). The former benefit from easiness of precise control of such model membrane parameters as its molecular packing, lateral pressure and composition.

Consistent results have been found upon analysing the effect of various xenobiotics on both natural and model membranes. For example, the formation of transmembrane channels (pores) by polyene antibiotics (e.g. amphotericin B), causing cells lysis (De Kruijff and Demel, 1974), was confirmed by Langmuir monolayer experiments (Seoane et al. 1998, 1999), proving the existence of strong interactions between the antibiotic molecules and membrane sterols (mammalian—cholesterol and fungi—ergosterol), leading to the formation of surface complexes, being responsible for the formation of channels. Another example is the increase of membrane fluidity observed in cell lines after the administration of anticancer drug—edelfosine (Santa-Rita et al. 2004; Gajate and Mollinedo 2001), which was confirmed on model membranes, mimicking leukemic cells (Hąc-Wydro and Dynarowicz-Łątka 2010). Alteration of cell membrane fluidity was also reported for toxic elements, such as mercury (Garcia et al. 2005). Further studies using model membranes enabled to get insight into this issue more deeply (Broniatowski et al. 2010). Also, cyclodextrins were studied in Langmuir monolayers imitating biomembranes. Although cholesterol depletion from natural membranes by cyclodextrins has been well documented as reviewed in ref. Zidovetzki and Levitan 2007, experiments on model systems allowed to carry out research in a broader aspect and dispelled doubts regarding the ability of phospholipids complexation by cyclodextrins (Flasiński et al. 2010; Jablin et al. 2010). Another example concerns the competition between membrane sterols and phospholipids, observed both in vivo (Lange et al. 2009) and in vitro (Miñones et al. 2009) studies.

All these examples show that studies on artificial systems are very useful as they confirm findings observed in vivo and are complementary to research carried out on living organisms.

This paper is aimed at analysing the effect caused by new generation anticancer drugs—alkylphosphocholines (APCs)—on prostate tumour membrane. Alkylphosphocholines belong to second generation antitumour lipids (ATLs), originally derived from lysophosphatidylcholine, however, of simplified structure, in which the glycerol backbone is lacking and substituted for a simple alkyl chain (Unger et al. 1992), as exemplified by hexadecylphosphocholine (miltefosine) (Eibl and Unger 1990), octadecylphosphocholine, perifosine and erucylphosphocholine (Jendrossek et al. 2003). Alkylphosphocholines have been found to be effective on various tumours cell lines, including breast cancer (Clive et al. 1999), acute leukaemia, lymphoma, colorectal cancer, multiple myeloma (Vink et al. 2005; Li et al. 2010; Crul et al. 2002; Gajate and Mollinedo 2007), brain tumours (Rübel et al. 2006) and prostate cancer (Rudner et al. 2010).

It is well known that cellular membranes of healthy and cancer cells differ significantly. For example, studies on leukaemia cells showed that the cancerous membrane is more fluid as compared to that of normal cells, which results mainly from decreased total cholesterol and cholesterol-to-phospholipids molar ratio (Johnson and Robinson 1979) and increased percentage of unsaturated fatty acids acyl chains of the major membrane phospholipids (Agatha et al. 2001). Similar tendency was also observed for other cancerous cell lines (e.g. lung cancer) (Sok et al. 1999), however, for prostate (Freeman and Solomon 2004) the effect was opposite, i.e. malicious membrane was more rigid as it contained increased level of cholesterol.

This paper is pointed at studying the effect of two selected alkylphosphocholines, namely hexadecylphosphocholine (miltefosine, HePC) and erucylphosphocholine (ErPC), on model prostate cancerous membrane and on living cells. Investigations on model system were performed using the Langmuir monolayer technique (Gaines 1966), while studies on biological systems were performed on the prostate cancer cells derived from brain metastasis cells (DU 145).

## Experimental

### Materials

The investigated antitumour alkylphosphocholines, hexadecylphosphocholine (HePC) and erucylphosphocholine (ErPC), both of >99 % purity, were obtained from Avanti Polar Lipids and Aeterna Zentaris GmbH, respectively. The following lipids, 1-palmitoyl-2-oleoyl-*sn*-glycero-3-phosphocholine (POPC, >99 % purity, purchased from Avanti Polar Lipids) and cholesterol (≥99 % purity, purchased from Sigma-Aldrich) were used to prepare model prostate cancerous membrane. All the studied substances were kept in closed bottles in the freezer and used without further purification.

### Langmuir Monolayer Studies

The spreading solutions for Langmuir monolayers experiments were prepared by dissolving appropriate weights of substances in spectroscopic grade chloroform/methanol (9:1 v/v) mixture. Langmuir monolayers were obtained by spreading an aliquot of the above-mentioned solutions (concentration of ca. 0.5 ± 0.2 mg/mL) with a Hamilton microsyringe (precise to ±2 µL) onto the surface of ultrapure water (resistivity ≥18.2 MΩ cm, pH 5.6, produced by the Millipore system). Mixed solutions were obtained by mixing proper volumes of respective stock solutions. The film compression (with the barrier speed of 50 cm^2^/min) was initiated 5 min after spreading to ensure total evaporation of the solvent. The surface pressure-area (*π*–*A*) isotherms were obtained with a 611 Langmuir–Blodgett trough (Coventry, U.K.) (total area = 600 cm^2^) placed on an anti-vibration table. Surface pressure was measured within 0.1 mN/m using a Wilhelmy plate made from ashless chromatography paper. Although using a filter paper may provoke errors due to the adsorption of film molecules, and therefore other materials like glass (Petelska and Figaszewski 2009) or platinum plate (Juskowiak and Swiatkowska 2013) are also applied, however, for the investigated molecules this was not the case. The temperature of the aqueous subphase was held constant to 37 ± 0.1 °C) by a circulating water system from Julabo.

The *π*–A isotherms were recorded for binary cholesterol/POPC model membrane as well as for ternary cholesterol/POPC/APCs monolayers of various drug concentration. Each experiment was repeated two or three times to ensure high reproducibility of the obtained isotherms to ±1 Å^2^. The mean area per molecules (*A*_12_) values were determined from the experimental *π*–*A* curves, and the compression modulus (*C*_S_^−1^) as well as the excess free energy of mixing (Δ*G* ^Exc^) values were calculated.

### Biological Studies

#### Cell Lines

DU-145 cells (the prostate cancer cells derived from brain metastasis) were cultured in RPMI 1640 medium supplemented with 10 % of foetal calf serum, 100 U/ml penicillin–streptomycin solution, 10 mM HEPES, 1 mM sodium pyruvate and 4.5 mg/ml glucose (Sigma-Aldrich, Steinheim, Germany) and conducted in a incubator at 37 °C and 5 % CO_2_.

#### Drug Treatment

Cells were incubated with staurosporine and APCs at a concentration of 12.5, 25 and 50 µM. Two different incubation times were applied (24 and 48 h) to observe time-dependent response. Two types of control cells were prepared: the negative one (cells untreated by any kinds of additional substances) and the positive one (cells treated by staurosporine at a concentration of 100 µM).

#### Determination of Cell Death

Cell death was analysed by fluorescence microscopy (Olympus BX51). Combined staining of the cells with Annexin V FITC to identify cells in which apoptosis has already been started and propidium iodide PI (Annexin V-FITC Apoptosis Detection Kit, Sigma-Aldrich) for the detection of necrotic cells was applied. Annexin V FITC and PI-positive cells were observed using U-MNB2 (excitement 470–490 nm) and U-MNG2 (excitement 530–550 nm) filters, respectively. Apoptotic and necrotic cells were quantified by cell counting in a population of approximately 10,000 cells. Each experiment was repeated three times independently. The Mann–Whitney U test was used to compare differences between groups (*p* < 0.05). Statistical analysis of the obtained data was performed using the Statistica software, version 8.0.

## Results and Discussion

### Influence of Alkylphosphocholines on Model Prostate Cancerous Membrane

**Fig. 1:**
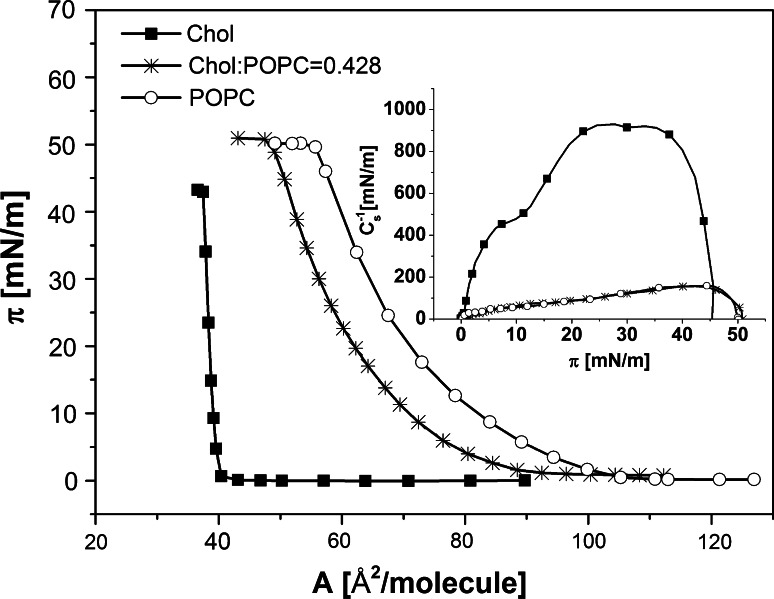
Surface pressure (*π*)–area (*A*) isotherms for binary cholesterol/phosphatidylcholine model of prostate cancer membrane and for pure components (cholesterol and POPC). Inset: compression modulus (*C*_s_^−1^)–surface pressure (*π*) *plot*

The model of cancerous prostate membrane was prepared based on ref. Calderon et al. 1991 by mixing cholesterol and POPC in the proportion of 0.428. Fig. 1 shows the pressure/area isotherm for a model membrane. For the purpose of comparison, the isotherms for its constituents (cholesterol and POPC) have also been incorporated.

**Fig. 2:**
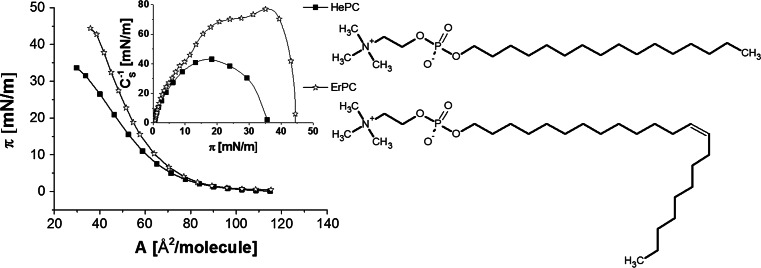
Surface pressure (*π*)–area (*A*) isotherms of HePC and ErPC spread at the air/water interface. *Inset*: compression modulus (*C*_s_^−1^)–surface pressure (*π*) dependencies

The *π*/*A* isotherms for cholesterol and POPC are in agreement with those reported in literature (see for example Miñones et al. 2009; or Cadena-Nava et al. 2006 for cholesterol and Yun et al. 2003 for POPC). The isotherm for model membrane is of a very similar shape to that for pure POPC, however, it is shifted towards smaller areas. The compression modulus, defined as *C*_S_^−1^ = −A (d*π*/d*A*), wherein *A* is the area per molecule at a given surface pressure *π* (Davies and Rideal 1963), has been calculated from the isotherm datapoints in order to characterise the physical state of the investigated monolayers (Figs. 1, 2, insets). The identical *C*_S_^−1^ values for pure POPC and mixed chol:POPC prove that both films are exactly of the same fluidity. The maximum *C*_S_^−1^ value reaches 160 mN/m, indicating liquid-condensed (LC) state of their monolayers. Cholesterol, on the other hand, forms very condensed, solid-type films. The investigated antitumour phosphocholines have already been studied in our former paper (Wnętrzak et al. 2013) and were found to form liquid monolayers (Fig. 2), which become more condensed (higher maximum *C*_s_^−1^ values) and more stable (higher values of collapse surface pressure, *π*_coll_) upon increasing of the hydrocarbon chain length of the drug molecule.

**Fig. 3:**
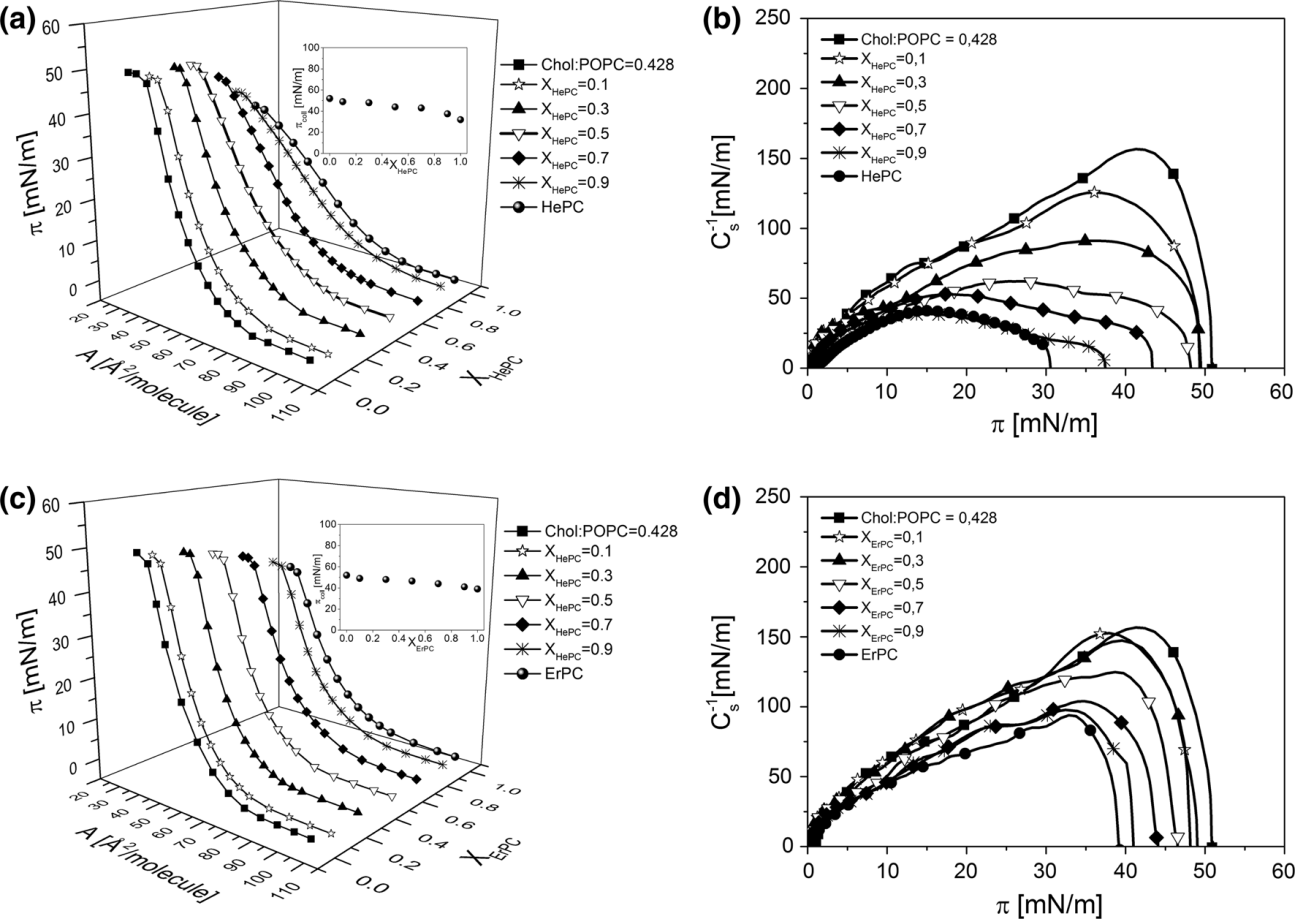
Surface pressure (*π*)–area (*A*) isotherms for Chol/POPC/APCs systems (**a**, **c**) and respective compression modulus (*C*_s_^−1^)–surface pressure (*π*) dependencies (**b**, **d**)

In order to get deeper insight into the effect of the investigated drugs on model cancerous prostate membrane, the investigated APCs were added into a monolayer mimicking model cancerous prostate membrane in various concentration (*X*_APC_ = 0; 0.1; 0.3; 0.5; 0.7; 0.9, 1). The surface pressure (*π*)–area (*A*) isotherms are shown in Fig. 3.

**Fig. 4:**
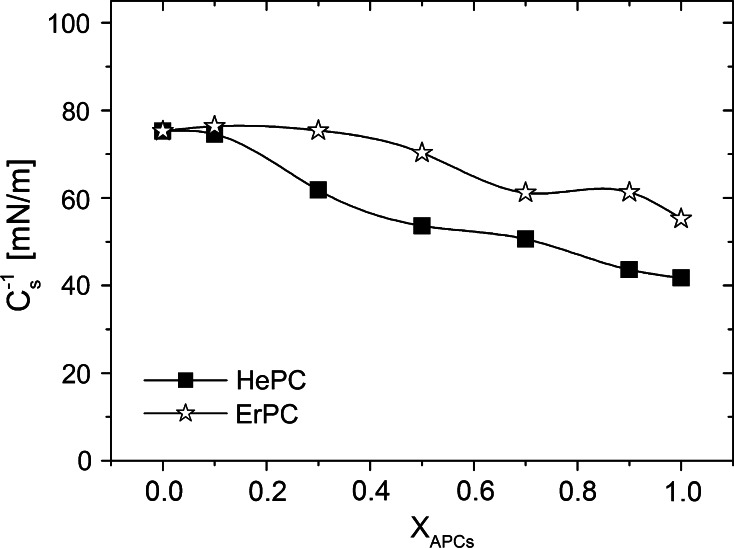
Decreases of the compression modulus values of the model cancerous prostate membrane caused by the investigated drugs versus mixed film composition at *π* = 15 mN/m

The isotherms for mixed monolayers lie in-between the isotherms for pure APC and model membrane. From the *C*_s_^−1^ versus *π* plots, it is seen that the incorporation of APC molecules to cholesterol/POPC monolayer decreases the compression modulus values. Fig. 4 confirms that both investigated drugs induce a fluidizing effect on a model membrane, HePC being more effective in this aspect as compared to ErPC.

For a deeper analysis of the effect of APCs on model cancerous prostate membrane, qualitative and quantitative parameters of interactions (Dynarowicz-Łątka and Kita 1999) have to be estimated. In the first step, the mean molecular areas (*A*_123_) have been determined from the experimental isotherms and compared with those resulting from ideal behaviour, calculated from the following equation:

1 $$A_{123}^{\text{id}} = A_{12} (X_{1} + X_{2} ) + A_{3} X_{3}$$

wherein *A*_12_ is the mean area per molecule in the mixed cholesterol/POPC film mimicking the cancerous prostate model membrane, *A*_3_ is the mean molecular area of the respective APC in its pure film at a given surface pressure and *X*_1_, *X*_2_ and *X*_3_ correspond to the mole fractions of components 1, 2 and 3 in the mixed film.

**Fig. 5:**
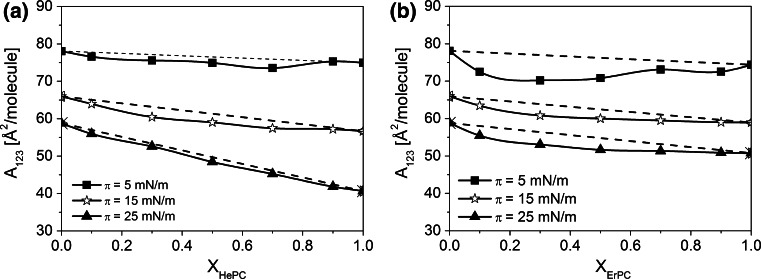
Mean molecular area (*A*_123_) versus mixed film composition (*X*_APCs_) *plots* for mixtures of HePC (**a**) and ErPC (**b**) with the investigated model membrane at different constant surface pressures

The non-linearity of the mean molecular areas versus composition plots (Fig. 5) proves miscibility and nonideal behaviour of the investigated mixed systems. Miscibility between film molecules is additionally confirmed by the collapse pressure values, which change with film composition as illustrated in Fig. 3, insets. The observed deviations in *A*_123_ versus composition dependencies are negative, indicating that the investigated drugs have the affinity to the model membrane.

**Fig. 6:**
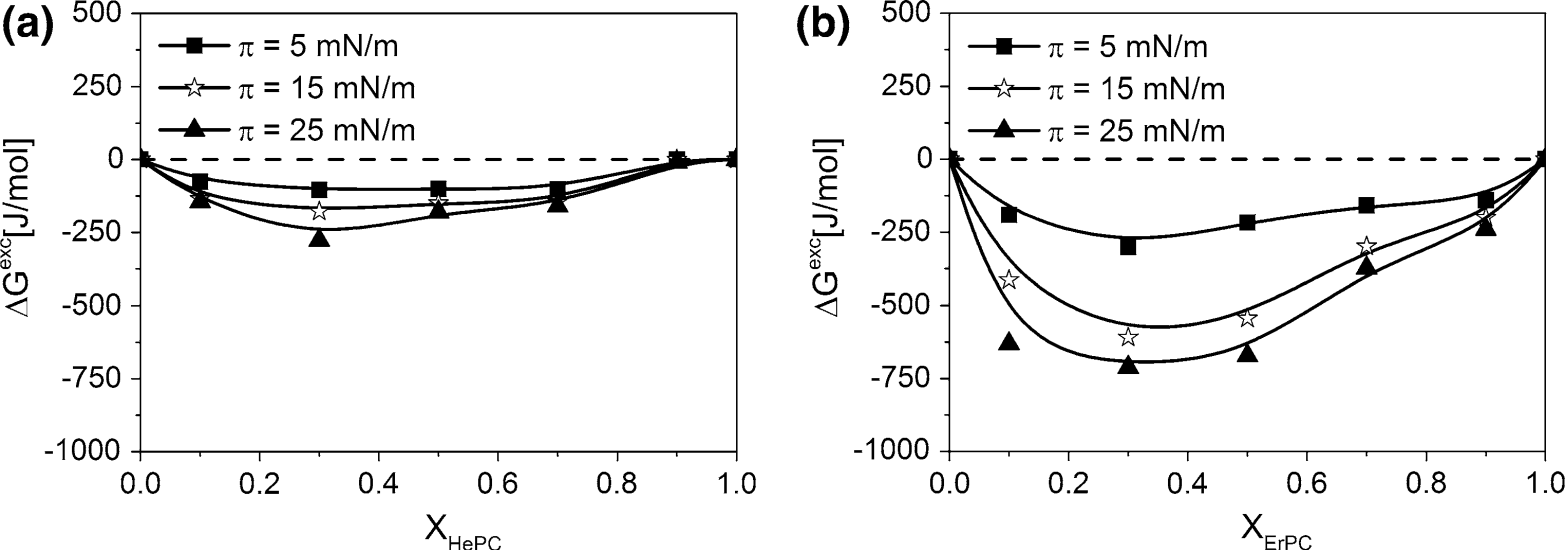
Excess free enthalpy of mixing (Δ*G* ^exc^) versus mixed film composition (*X*_APCs_) *plots* for mixtures of HePC (**a**) and ErPC (**b**) with investigated model membrane at different constant surface pressures

To quantify the drug-membrane interactions, the excess free energy of mixing (Δ*G* ^Exc^) values has been calculated using Eq. 2:

2 $$\Delta G^{\text{Exc}} \; = N\;\int\limits_{0}^{\pi } {\left( {A_{123} \; - \;(X_{1} \; + \;X_{2} )A_{12} \; - \;X_{3} A_{3} } \right)\;{\text{d}}\pi }$$

where *N* is the Avogadro’s number, *A*_123_ is the mean area per molecules in ternary film. The results of the foregoing calculations are shown in Fig. 6.

The negative values of Δ*G* ^Exc^ suggest stabilising—from thermodynamic point of view—effect of both investigated APCs on the monolayer mimicking model membrane, and the presence of stronger interactions between the components in ternary mixtures (1–2–3) as compared to those existing in cholesterol/POPC model membrane (1–2). Therefore, it can be concluded that the incorporation of APCs is favourable for binary cholesterol/POPC monolayer, serving as a model of cancerous prostate membrane. Additionally, highly negative values of Δ*G* ^Exc^ for ErPC-containing films prove that the interaction with model membrane are stronger (ca. 3-times) for ErPC versus HePC.

### Induction of Apoptosis by APCs: Biological Studies

**Fig. 7:**
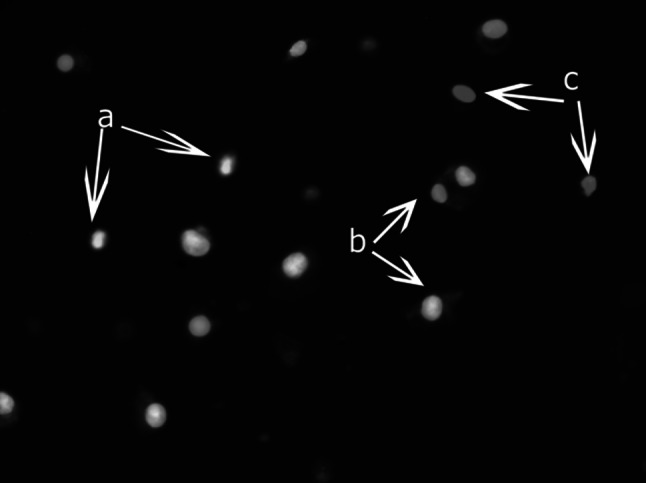
Induction of apoptosis and necrosis in Du145 cells incubated with ErPC was evaluated by fluorescence microscopy (combined staining with Annexin V FITC and PI). The image shows cells undergoing early-stage apoptosis *a* *green* fluorescence; late-stage apoptosis *b* *green* and *red* fluorescence, and necrotic cells *c* *red* fluorescence

Programmed cell death (apoptosis) is a physiological process that occurs by elimination of unnecessary or defective cells. In contrast to necrosis, apoptosis is desirable and does not cause inflammation state. This process determines the proper functioning of the living organism (Elmore 2007). Apoptosis can be induced spontaneously (i.e. as a homeostatic mechanism) (Youlcu et al. 2008) or by external factors (e.g. drugs or irradiation) (Rübel et al. 2006). One of the earliest change observed in the cell in the apoptotic pathway is disorder of cell membrane asymmetry, which occurs as a result of displacement of the phosphatidylserine (PS) from the inner side of the plasma membrane to the surface (Williamson and Schlegel 2002). Annexin V labeled with fluorescein isothiocyante FITC has a high affinity for PS; therefore, it binds to cells with exposed PS on the surface and can be used to the determination of an early stage of apoptosis. On the other hand, propidium iodide PI penetrates only into dead or damaged cells—viable cells with intact membrane exclude PI. Fluorochrome PI is an intercalating agent. When the cell membrane is disrupted, PI penetrates into the cell and binds to the DNA and RNA; therefore, already dead cells fluoresce red (van Engeland et al. 1998). On this basis, the identification of necrotic or late apoptotic cells is possible (Fig. 7).

**Fig. 8:**
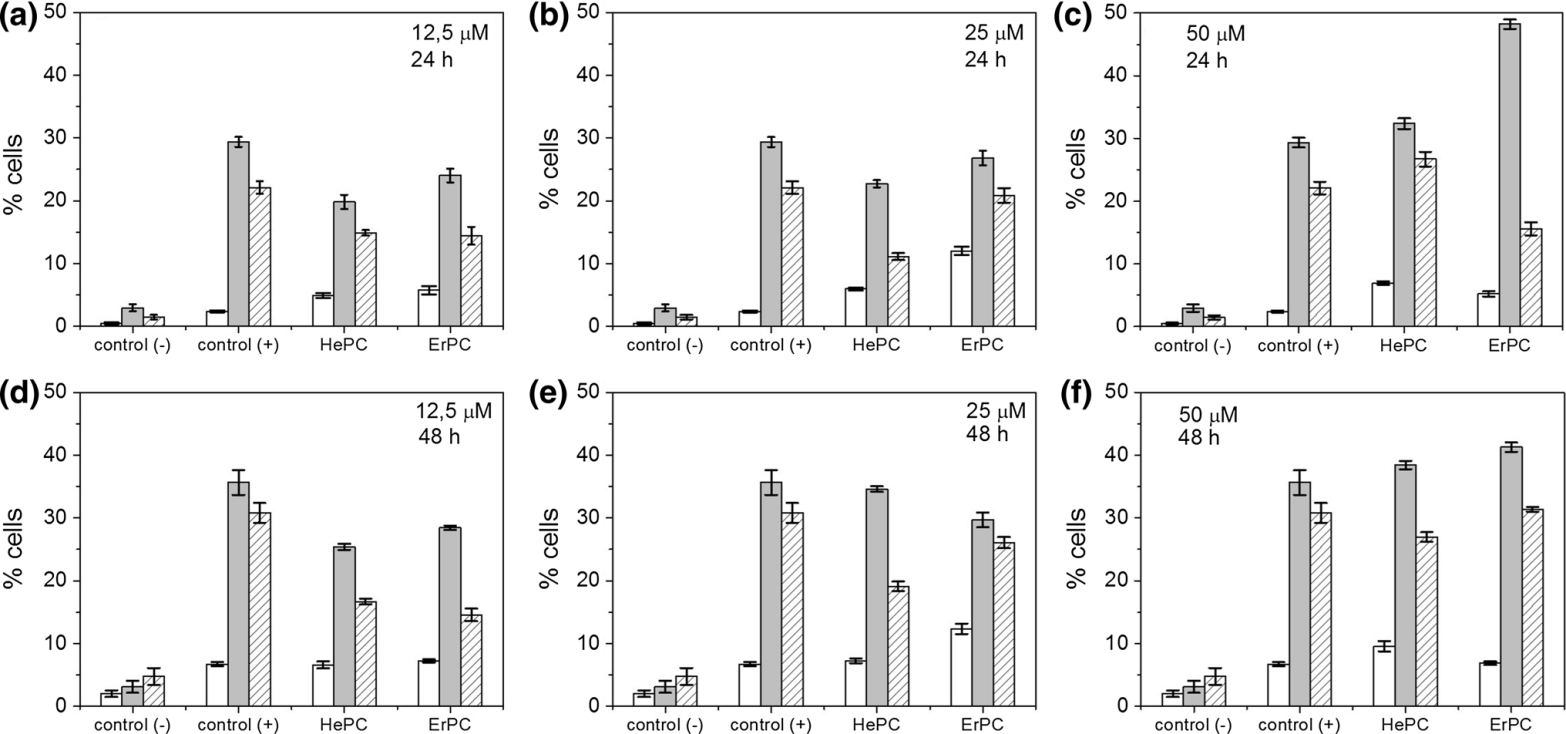
The amount of necrotic (*white bars*), early (*grey bars*) and late (*stripped bars*) apoptotic cells was determined 24 h (**a**–**c**) and 48 h (**d**–**f**) after APCs treatment and expressed in percent of total cells (= 100 %) for different drug concentrations (12.5, 25 and 50 µM); the results for the untreated control group [control (−)] and for the staurosporine- treated group [control (+)] are shown for comparison

The number of apoptotic and necrotic cells was counted from each image (showing approx. 10,000 cells) and normalised to the total cell number. As a calibration, both untreated samples (negative control culture) and cells incubated with staurosporine—an antibiotic commonly used as an initiator of apoptosis (positive control culture) were used. Dose-dependent and incubation time-dependent results for each analysed group are shown in Fig. 8.

As can be seen in Fig. 8, in a untreated sample the population of apoptotic and necrotic cells after 24 h was small and the fraction of living cells was approx 94 %. Drug treatment caused a noticeable increase of the apoptotic cells number with relatively low percentage of cells in necrotic stage. After treatment with the lowest drug concentrations (12.5 µM), the achieved results were comparable: necrosis at 5 %, early apoptosis at 20–25 % and late apoptosis at approximately 14 %.

**Fig. 9:**
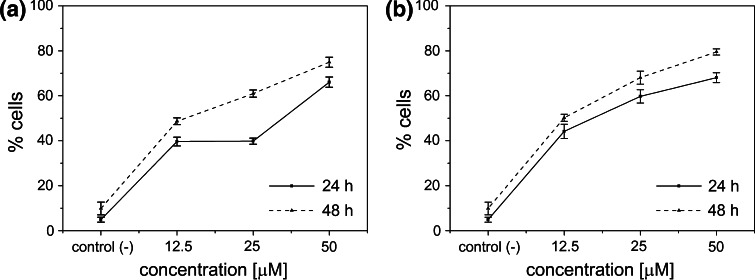
The percentage of apoptotic cells (together in early and late stages) as a function of drug dose compared to untreated control group [control (−)] for two incubation time presented for each drug separately: **a** HePC, **b** ErPC

Figure 8 reveals that doubling of the dose of HePC from 12.5 to 25 µM has independently changed the percentage of cells in early and late stages of apoptosis, however—when these cells were considered altogether—there were no changes in cells mortality by the apoptotic pathway (Fig. 9). In contrast, an increasing concentration of ErPC led to the increase of the percentage of apoptotic cells (late and early stages), which is shown in Fig. 8. An incubation for 24 h with the highest drug dose—50 µM—caused a significant increase of the apoptotic cell number. The highest percentage of apoptotic cells was observed in population treated with 50 µM (visible especially in ErPC-treated samples).

For longer incubation time—48 h—the increase of the amount of apoptotic and necrotic cells was observed in relation to the shorter exposure time (both in untreated and APCs-treated cells). For APCs-treated cells, the number of cells undergoing apoptosis was as follows: early apoptosis ~27 at.%, late apoptosis~26 at.% for 12.5 µM, early apoptosis ~30 at.%, late apoptosis ~26–29 at.% for 25 µM and early apoptosis ~37–40 at.%, late apoptosis ~26–30 at.% for 50 µM. Likewise, the population of necrotic cells has changed only slightly (from 5 to 7 %). All the obtained results for each studied drug dose and incubation time were statistical significant (*p* < 0.05) in comparison to the control untreated group.

## Conclusions

Model studies carried out with the Langmuir monolayer technique unambiguously prove that both investigated APCs interact with a monolayer mimicking prostate cancer membrane. The observed effect is two-fold. Namely, the thermodynamic analysis based on Δ*G* ^Exc^ calculations evidences for the existence of strong drug-membrane interactions. The magnitude of the Δ*G* ^Exc^ values proves that ErPC has a higher affinity to a model membrane as compared to HePC. Therefore, one could expect that ErPC has more effective therapeutic effect. Moreover, both studied drugs were found to fluidize the model membrane. A plethora of studies performed on living systems, reviewed in ref. Tekpli et al. 2013, confirm that a change of cellular membrane elastic properties frequently leads to apoptosis. Indeed, biological studies confirmed that in Du-145 cell line APCs cause cell death primarily by apoptosis while necrotic cells constitute only a small percentage of APC-treated cells. An application of combined staining with Annexin V FITC and propidium Iodide allowed for a rapid, simultaneous detection of cells in early apoptotic stage (still alive) and late apoptosis stage (already death). To the best of our knowledge, this combination of biochemical stainings was applied for the first time in Du-145 cell death detection after APCs treatment. The results indicate an increase of therapeutic effect, which is associated with prolonged drug incubation time and the use of higher drug concentration. It was proved that ErPC is the most potent drugs of the investigated APCs, inducing apoptosis in DU-145 cells, which was inferred from the Langmuir monolayer studies. Additionally, investigations on cell lines prove that ErPC is a fast-acting drug in comparison with HePC. Just 24 h incubation with 50 µM ErPC inducted apoptosis in approx. 70 % of the population.
